# Potential Positive Effects of COVID-19 on Cancer Care: A Window of Opportunity

**DOI:** 10.3389/ijph.2022.1604394

**Published:** 2022-02-25

**Authors:** Emmanouil Damilakis

**Affiliations:** Department of Pharmaceutical Sciences, University of Basel, Basel, Switzerland

**Keywords:** COVID-19, vaccination, telemedicine, environmental risk factors, cancer, drug research, mRNA

**The IJPH series “Young Researcher Editorial” is a training project of the Swiss School of Public Health**

Healthcare services are heavily burdened by the coronavirus (COVID-19) pandemic that began in 2019. The pandemic placed extraordinary strain on cancer drug supply chains and the virus’s disruption of cancer care is well-documented [1, 2]. Despite this disruption, the pandemic may also have opened opportunities to improve cancer care. Identifying opportunities and challenges to improve cancer prevention, diagnosis and treatment should be a priority for governments, health and regulatory authorities, research scientists, drug manufacturers, and societies.

To meet the challenges of future pandemics and other emergencies, the European Medicines Agency (EMA) established the “EU Executive Steering Group on Shortages of Medicines Caused by Major Events” in March 2020. The Steering Group put measures in place to avoid or prevent disruptions in drug supplies during emergencies in European Union member states [3]. Surveys taken by members of the European Society of Oncology Pharmacy (ESOP) revealed that, during the COVID-19 pandemic, half the oncology pharmacists in Europe experienced shortages of anti-cancer medications [4]. Understanding why some vital drugs were in short supply could help governments and health systems prevent such shortages in the future, as well as help manufacturers improve drug supply chains.

Developing drugs and vaccines is commonly an arduous, costly, 12–15-years process [5], but since the onset of the COVID-19 pandemic, academia and industry reached an unprecedented level of cooperation. Experts around the world and across disciplines have joined forces to learn more about the disease and how to control it, and collaborators have developed, validated, and deployed COVID-19 medications at record speed. In less than a year, several vaccines proceeded through phase 1–3 testing and were approved by regulatory authorities for human use [6]. This accomplishment has proven that accelerating regulatory processes could reduce approval timelines for essential vaccines and drugs. This knowledge can be applied to design faster, better optimized approval processes for life-threatening diseases like cancer, which could help save the lives of thousands of patients globally.

Vaccination is an effective way to control infectious diseases. While messenger RNA (mRNA) technology has been a point of interest in the past, mRNA vaccines against COVID-19 were the first of this kind to receive approval by the Food and Drug Administration (FDA), the EMA and other regulatory authorities and now play a central role to fight the pandemic [7]. This massive leap in vaccine discovery has put mRNA technology in the spotlight, encouraging the exploration of its potential uses against cancer. Drug manufacturers are currently conducting clinical trials using mRNA technology to treat different types of cancer with promising preliminary results. For example, a study showed that vaccination is potent immunotherapy in patients with unresectable melanoma [8]. Further research is needed to find out whether this technology enables the treatment of cancers which cannot be addressed adequately with existing drugs.

Another consequence of the COVID-19 pandemic that should be leveraged to improve future cancer care is the increased use of digital communication. Emphasis should be placed on cancer prevention using digital media. Online training has the potential to help people better understand cancer and take preventive lifestyle measures so they can minimize the possibility of developing malignancies. Cancer patients who live in remote areas, and those who do not have relatives or friends who can accompany them to an appointment can now take advantage of telemedicine. However, there are also issues that need to be addressed. Many older patients are not familiar with digital information and communication. Societies must ensure that these people have access to digital technology and receive relevant training tailored to their needs.

To control the spread of the virus, governments have decided to restrict the movement of people. Travel restrictions have reduced polluting human activity, which in turn reduced environmental carcinogens [9]. Studies have shown that long-term exposure to environmental pollutants can induce lung cancer and increase cancer risk in specific anatomical locations [10]. It is reasonable to assume that air quality levels will return to pre-pandemic levels as soon as movement restrictions are lifted. Governments should motivate citizens to continue living in an environmentally friendly way, to decrease the likelihood of cancer, as well as to protect the planet.

We can turn this pandemic into an opportunity for cancer care. COVID-19 crisis showed that strategies to improve drug supply chains should be developed, regulatory processes for essential vaccine and drug approval can be accelerated, novel cancer treatments should be supported, digital communication should be better utilized for cancer prevention and cancer treatment and man-made environmental carcinogens can be reduced in order to decrease cancer risk. It is conceivable that COVID-19—despite the public health and societal impact—played a key role in unlocking scientific advances and new collaborations to combat cancer and possibly other diseases that have plagued humans since the dawn of time.
